# Adolescent Social Anxiety, School Satisfaction, Family Emotional Support, and School Absenteeism: Findings from Young-HUNT3 and Norwegian National Education Data

**DOI:** 10.3390/jcm13092547

**Published:** 2024-04-26

**Authors:** Malik D. Halidu, Yasuhiro Kotera

**Affiliations:** 1 Faculty of Nursing and Health Science, Nord University, 7600 Levanger, Norway; 2 Faculty of Medicine and Health Sciences, University of Nottingham, Nottingham NG7 2RD, UK; yasuhiro.kotera@nottingham.ac.uk; 3 Center for Infectious Disease Education and Research, Osaka University, Suita 565-0871, Japan

**Keywords:** family emotional support, social anxiety, school satisfaction, school absenteeism, Norwegian adolescent

## Abstract

**Background**: Adolescents grappling with social anxiety may experience poor school satisfaction, resorting to school-related avoidance behaviors, exemplified by absenteeism, as a coping mechanism. Understanding the role of family support in alleviating the adverse effects of social anxiety on school satisfaction is imperative for fostering supportive educational settings. Although there is literature regarding how school satisfaction promotes positive adolescent outcomes, empirical knowledge on the interrelation between social anxiety, school satisfaction, and family emotional support is limited. This study investigates the association between social anxiety, family emotional support, school satisfaction, and school absenteeism within the theoretical framework of the stage-environment fit theory to offer insight into how family emotional support can moderate the influence of social anxiety on school-related outcomes. **Methods:** Utilizing a population-based sample of 1861 upper secondary school pupils from the Trøndelag Young Health study “Young-HUNT3 study”, we employed an index of moderated mediation to examine the role of family emotional support in moderating the association between social anxiety and school-related avoidance behavior related to school satisfaction. **Results:** Family emotional support had moderated mediation association for school absenteeism (β = 0.128, 95% CI 0.019, 0.278) and extracurricular activity (β = −0.003, 95% CI −0.008, −0.000). **Conclusions**: This urges further investigation into the specific mechanisms and individual differences influencing these relationships, aiming to deepen our understanding of adolescents’ experiences and inform comprehensive strategies for promoting their well-being within school communities.

## 1. Introduction

Focusing on health and quality education is paramount in this era of pursuing Sustainable Development Goals (SDGs) [1,2,3,4,5]. Adolescents grappling with social anxiety, characterized by fear and discomfort in social situations [6], often exhibit avoidant behaviors [7], including absenteeism, poor school satisfaction, and spillover effects on non-academic activities such as sports and other team-based extracurricular activities [8,9]. Research indicates that social anxiety is a prevalent mental health issue among students [10]. The inherently social nature of schooling and team-based performance of extracurricular activities may self-evidently contribute to school absenteeism and avoidance of group-based activities among socially anxious adolescents [11]. School absenteeism, defined as “the emotional upset of the likelihood of attending school or missing classes regularly” [12], is a significant challenge in educational systems worldwide [13]. Additionally, social anxiety is acknowledged as a cross-cultural phenomenon [14], while absenteeism is recognized as a complex issue with global implications [15]. The global nature of these challenges emphasizes the need for comprehensive research and interventions on an international scale in order to improve overall school success.

The relationships between social anxiety and school absenteeism (SAB) remain fragmented [16,17]. This gap persists amid inconsistencies in findings [16] and a lack of robust theoretical perspectives [18,19]. To address this, our study is anchored in the stage-environment fit theory [20,21], a foundational framework in developmental psychology. This theory posits that individuals progress through distinct developmental stages during their educational journey, each characterized by unique challenges and tasks.

Adding a novel dimension, our study incorporates “functional contextualism” to underscore the importance of understanding behavior within its context [22]. This approach justifies our exploration of how family culture/atmosphere, as a contextual influence, may shape individual experiences within the educational setting. Emphasizing the interconnectedness within families and the impact of cultural dynamics, our study integrates family systems theories [23,24] and cultural psychology [25,26] within the stage-environment fit framework. This theoretical synthesis aims to uncover how family culture/atmosphere contextualizes the mechanisms between psychological state and interrelationship with behavioral outcomes, providing insights into the complex relationships between social anxiety, school satisfaction, absenteeism, and team-based extracurricular activity.

While the stage-environment fit theory remains central, functional contextualism, family systems theories, and cultural psychology collectively enhance our exploration, offering additional layers of understanding without diluting the primary theoretical framework. This integrated approach provides a nuanced examination of the interplay between social anxiety, school satisfaction, and absenteeism during adolescence, addressing critical gaps in the existing literature.

In Norway, education is a legislated social right that guarantees equal rights to education regardless of the place of residence, gender, or social background [25]. Education from primary and secondary levels is free. Education is mandatory from primary to lower secondary school [27,28]. School enrollment is an individual choice at the upper secondary level, although almost everyone who completes compulsory primary schooling enrolls [28]. At the upper secondary level, pupils are awarded two grades—one that reflects their academic grades, and another based on their attendance rate, utilizing a rule set at a 10–15% range granted by school heads to pupils [29]. High absenteeism rates are a risk factor for dropout from upper secondary school [30,31]. There is a consensus among stakeholders in Norway that the current rates of absenteeism are unacceptable, and arriving at optimal solutions is imperative [29]. According to the annual Organization for Economic Co-operation and Development 2023 (OECD) [26], although there is a general increase in upper secondary attainment across OECD countries, 14% of young adults across the OECD still leave school without an upper secondary qualification. In Norway, the share is 17%, higher than the OECD average. Improving upper secondary education completion rates and lowering school absenteeism have been associated with student school satisfaction [32,33]. Our study primarily focuses on adolescents, defined as high school students, to examine the influence of home and school environments on the interplay between social anxiety and school outcomes. While we hypothesize a unidirectional relationship, we acknowledge the possibility of bidirectional influences between the variables under investigation. We conduct a systematic analysis of (a) the relationships between social anxiety and two key school outcomes—absenteeism and participation in team-based extracurricular activities, (b) the mediating role of school satisfaction, and (c) the moderating role of family emotional support on the links between social anxiety, school satisfaction, school absenteeism, and participation in a team-based extracurricular activity.

### 1.1. Theoretical Framework

Functional contextualism [22], associated with acceptance and commitment therapy (ACT), focuses on behavior’s function within a context, emphasizing its dynamic relationship with the environment. It emphasizes understanding behavior by considering its function or purpose within a specific context. Key tenets focus on the dynamic relationship between behavior and its environment, recognizing that behavior cannot be fully grasped in isolation. In developmental psychology, the stage-environment fit theory explores individuals’ interaction with evolving developmental stages. Integrating functional contextualism within stage-environment fit underscores a shared emphasis on dynamic context [20]. This fusion provides a nuanced perspective on how adolescents navigating educational stages with social anxiety shape behavioral outcomes. It allows for hypotheses exploring the evolving function of anxiety-influenced behaviors across stages, enriching the theoretical foundation for the study. Thus, hypothetically, we posit that social anxiety is negatively linked to school satisfaction, asserting that social anxiety dynamically shapes perceptions, impacting satisfaction levels during the educational journey.

### 1.2. Linking Social Anxiety to School Satisfaction

As discussed above, the stage-environment fit theory explicates the building blocks to conceptualize the dynamic interaction between individuals and their educational stages. In particular, there is an association between social anxiety and school satisfaction, a subjective and cognitive assessment of the perceived quality of school life. Social anxiety, recognized as a psychological state, may contribute to the constellation of factors that disrupt perceived fit within an educational stage. The theory emphasizes that challenges in social interactions contribute to a sense of mismatch as individuals progress through stages, influencing perceptions of fit and satisfaction [20,21]. Thus, positive experiences in social situations may interfere with excessive worry and contemplation, likely due to increased levels of internal self-focused attention that socially anxious individuals exhibit [34]. Thus, hypothetically, one possible association is that social anxiety is negatively associated with school satisfaction, albeit social anxiety may dynamically relate to perceptions impacting school satisfaction as a temporal sequence (i.e., longitudinal bidirectionality) throughout the educational journey.

### 1.3. Unraveling the Indirect Role

The stage-environment fit theory introduces the concept of “fit” or “mismatch”, describing alignment or misalignment between individuals and their academic environment at each stage [20,21,35]. This perspective acknowledges the evolving nature of fit during different educational phases. Social anxiety, viewed as a factor disrupting perceived fit within an educational stage, is expected to contribute to a sense of mismatch as adolescents progress through stages [21,36,37]. Here, we introduce school satisfaction as an intermediary, aligning with the stage-environment fit theory’s tenet, which states that individual perceptions mediate the impact of psychological states on behavioral outcomes. This indirect association illustrates the nuanced interplay between social anxiety, perceptions of fit, and resultant behavior.

### 1.4. Theoretical Integration within the Stage-Environment Fit Framework

Integrating family systems theories and cultural psychology within the stage-environment fit framework provides insights into how family dynamics and cultural influences shape academic experiences [35,38]. Family systems theories emphasize interconnectedness within families, influencing perceived fit in educational environments [39,40,41]. Cultural psychology underscores how shared beliefs and practices create a unique atmosphere, impacting experiences in different educational stages [42,43]. This comprehensive framework highlights the multifaceted nature of fitting within different educational stages, where family dynamics and cultural influences dynamically shape perceptions, contributing to overall well-being and behavioral outcomes. Thoits’ study [44] suggests that family emotional support buffers psychological distress. Also, one study [45] shows that family support is relevant to pupil satisfaction with school. Therefore, we propose including family emotional support as a contextual moderator. Our hypothesis suggests that it buffers the indirect impact of social anxiety on absenteeism (and involvement in extracurricular activities) via school satisfaction. This comprehensive framework highlights the multifaceted nature of fitting within different educational stages, where family dynamics and cultural influences dynamically shape perceptions, contributing to overall well-being and behavioral outcomes. In sum, three main hypotheses were established.

**Hypothesis** **1 (H1).** *Social anxiety is negatively associated with school satisfaction.*

**Hypothesis** **2a (H2a).** *Social anxiety is positively associated with school absenteeism through reduced levels of perceived school satisfaction.*

**Hypothesis** **2a (H2a).** *Social anxiety is negatively associated with extracurricular activities through reduced levels of perceived school satisfaction.*

**Hypothesis** **3a (H3a).** *The indirect association between social anxiety and school absenteeism through perceived school satisfaction is moderated by the level of family emotional support.*

**Hypothesis** **3b (H3b).** *The indirect association between social anxiety and extracurricular activity through perceived school satisfaction is moderated by the level of family emotional support.*

## 2. Materials and Methods

### 2.1. Data Sample

Data from the Trøndelag Young Health study (Young-HUNT3; 2006–2008) comprise lower and upper secondary school adolescents aged 13–19 living in the Trøndelag county of Norway. Despite being nearly 15 years old, the dataset remains uniquely valuable due to its rare insights and specific conditions not replicated in newer datasets. Its age is beneficial for providing a historical baseline and is crucial for future longitudinal studies. No comparable recent datasets exist that can adequately replace the depth and specificity of the data we have. We have applied modern analytical techniques to ensure robustness in our findings, highlighting these data’s ongoing relevance and scientific contribution to our field. We linked data from the Young-HUNT3 questionnaire to the Norwegian National Education Database (NUDB) with a unique 11-digit national personal identification number assigned to each Norwegian resident. Of the 4357 upper secondary school pupils invited for the Young-HUNT3 survey, a response rate of 76.6% (3336 respondents) was attainted [46]. For this study, the sample contains the pupils who participated in Young-HUNT3 in 2008 at the upper secondary school level. A missing completely at random (MCAR) [47] was estimated to test whether a significant difference exists between the means of different missing-value patterns between school absenteeism, social anxiety levels, school satisfaction, and family emotional support. Based on Little’s MCAR test result, $\chi^{2}\left(7.31\right)=8,p>0.50$, this suggests that the dataset’s missingness is completely random. After a listwise deletion of missing variables, the final sample size was 1864.

### 2.2. Measures

#### 2.2.1. School Absenteeism (SAB)

SAB records were obtained from NUDB, indicating the number of days of absence of each participant over the academic year in which they participated in the Young-HUNT3 study. SAB is documented on the student’s ultimate diploma. The rules for the documentation are as follows: (1) A maximum of 10 days of absences (approved and unapproved) can be omitted from the diploma. (2) Absences attributed to non-chronic illnesses may only be omitted from the diploma if SAB endures for a minimum of three consecutive days. (3) Days missed because of authorized leave or chronic illnesses may be excluded, commencing from the first day of SAB, and erased from the diploma if the absence surpasses 10 days [48].

#### 2.2.2. Self-Reported Extracurricular Activities

Extracurricular activities, defined as after-school activities after school hours, was self-reported from the Young-HUNT3 study utilizing an index of 9 items. A sample item is “How often have you done/participated in the following activities in the past 12 months (e.g., team sports, endurance sports)?”. Each item was measured on a 4-point Likert scale (1 = never, 4 = several a week). A composite score was calculated as the sum of the scores of all nine items.

#### 2.2.3. Perceived Pupil School Satisfaction (SS)

School satisfaction, a subjective and cognitive appraisal of the perceived quality of school life, was evaluated using the school functioning scale from Young-HUNT3. The school-functioning statements were composed at the Norwegian Institute of Public Health and included in an earlier study of childhood abuse at the same institute [49]. A sub-dimension of the scale comprising six items covering satisfaction with school [9,50] was utilized. Individuals responded to a four-point scale ranging from “never” (1) to “very often” (4). The items include “*Look forward to going to school*” and “*Are satisfied with your test results*”. Cronbach’s alpha was 0.71. A mean composite score on the item’s higher scores indicates high levels of SS.

#### 2.2.4. Social Anxiety Symptom Index (SA)

Non-clinical levels of SA as perceived by adolescents were assessed from a self-reported Young-HUNT3 questionnaire based on six items, a shortened scale of the Social Phobia and Anxiety Inventory for Children (SPAI-C). The six items were selected from SPAI-C [50,51] and SPAI [52], using an item analysis approach [53,54]. The SPAI-C demonstrates good convergent validity [50] and is reliable and valid in adolescents [55]. Each participant self-rated on a five-point Likert scale. Each item was placed on an ordinal scale (1 = never, 5 = always), which was summed (range 6–30). Sample items were “*I feel anxious and don’t know what to do in an embarrassing situation*”, and “*I feel anxious when I am with others and have to do something while they watch me do it (e.g., be in a play, play music, sports [36,43])*”. Cronbach’s alpha was 0.85. For this study, the composite score was calculated as the average of the six items, with high scores indicating more symptoms.

#### 2.2.5. Perceived Family Emotional Support (PFS)

Self-reported PFS measures from Young-HUNT3 were assessed using four items selected from nine items included in a Resilience Scale for Adolescents (READ). Each item was rated on a five-point Likert scale, with questions like “In my family, we share views of what is important in life” and “I feel comfortable with my family”. In the current study, Cronbach’s alpha was 0.82. Items were reversed codeded.

#### 2.2.6. Control Variables

Guided by the relevant literature reviewed above, participants’ self-reported demographics and health-related variables such as gender, self-rated family financial status, self-rated health status, and class level (i.e., Year 1–3) from the Young-HUNT3 survey were used as control variables.

### 2.3. Analytical Procedure

Before the hypotheses were tested, a confirmatory factor analysis (CFA) model for the SA, SS, and PFS scales was fitted to ensure construct validity and justify calculating composite scores as the average of scale items. Next, the hypothesized associational moderated mediation model was estimated with bootstrapping. The R statistical software version 4.2.2 [56] was used to run the PROCESS macro (models 4 and 7) and to assess the statistical significance of the indirect and conditional indirect effects at differing levels of the moderator (i.e., Johnson–Neyman interval) based on a bias-corrected 95% confidence interval from 10,000 bootstrap samples [57]. The SA, PFS, and SS were mean-centred, with SA taken at 1SD below and above the mean. In addition, an index of the moderated indirect path was estimated to test the significance of the moderated indirect path, i.e., the difference in the indirect association across levels of the moderator (family emotional support) (see [58]). Significant associations are supported by the absence of zero within the bias-corrected confidence intervals.

### 2.4. Measurement Model and Common Method Bias

The measurement model (CFA) of the SA and SS constructs shows a satisfactory fit to the data (RMSEA = 0.075, CFI = 0.930, and SRMR = 0.042) and satisfies the cutoff of model fit indices (see Hu and Bentler [59]). In addition, the average variance extracted (0.492.3 for SA, 0.346 for SS, and 0.629 for PFS) was higher than the squared factor correlations. (r = 0.104, 0.105, 0.119). Thus, our measurement model fulfills this condition of discriminant validity. The reliability coefficients were acceptable, with Cronbach’s alpha of 0.85, 0.71, and 0.82 for SA, SS, and FPS, respectively. Hence, composites are justified and, thus, were created.

## 3. Results

Table 1 reports the descriptive statistics of means, standard deviation (SD), and distributional proportions for each variable in the study. Fifty-seven percent (N = 1063) of the sample was female. The class level of the sample was the first year (35.3%, N = 658), second year (40.5%, N = 1755), and year-3 (24.2%, N = 451). Regarding the family’s financial situation 68.7% (N = 1281) of the adolescents reported being equal as compared to others, 22.3% (N = 416) reported being better off financially compared to others, and 9.0% (N = 167) reported been worse off compared to others. In terms of self-reported health status, 10.8% (N = 201) of the sample reported being not good, 53.3% (N = 993) reported being good, and 35.9% (N = 670) reported being very good. The average SA score was 1.96 (SD = 0.72). The average SS score was 2.93 (SD = 0.49). The average SAB score was 7.55 (SD = 7.62). Regarding correlations among main study variables, SA is positively correlated with SAB (*r* = 0.055, *p* < 0.05), and negatively correlated with school satisfaction *(r* = −0.175, *p* < 0.05), and family emotional support *(r* = −0.098, *p* < 0.05) as shown in Table 2. The correlation coefficient range among the study variables was *r* = 0.01 to 0.30. The small size of correlations among predictor variables seems to suggest that the risk of multicollinearity was minimal.

**Figure 1:**
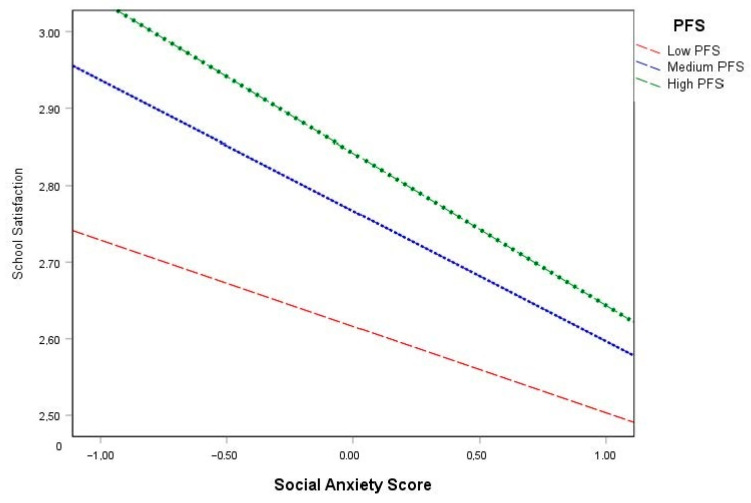
First-stage moderation.

Results of the associational moderated mediation analysis are provided in Table 3. The direct association between social anxiety and school-related activities was found not to be statistically significant for school absenteeism (*b* = −0.222, *p* > 0.05) and extracurricular activities (*b* = −0.014, *p* > 0.05). The association between social anxiety and school satisfaction was also conditional on levels of family emotional support for school absenteeism (*b* = −0.055, *p* < 0.05) and was statistically significant. In addition to estimating model parameters, Figure 1 visually depicts the interaction between social anxiety and family emotional support on school satisfaction. As shown in Figure 1, social anxiety was negatively associated with school satisfaction for all levels of family support, such that as social anxiety increased, school satisfaction decreased. However, as depicted by the steepness of the slopes, the negative relation between social anxiety and school satisfaction was largest in magnitude among adolescents characterized by low levels of family emotional support. Most notably, a formal test of associational moderated mediation based on the index term [58] revealed that family emotional support moderated the indirect association of social anxiety on school absenteeism (*b* = 0.128, 95% CI = 0.019, 0.278) and extracurricular activities (*b* = −0.003, 95% CI = −0.008, −0.000).

Further hypothesis tests were conducted to determine whether the conditional indirect association was statistically significant at values corresponding to low, moderate, and high values of family emotional support, as noted above (see Table 4). Results revealed that school satisfaction differed in the association between social anxiety and school absenteeism for adolescents with low family support (*b* = 0.216, 95% CI = 0.098, 0.388), moderate family emotional support (*b* = 0.322, 95% CI = 0.193, 0.500), and high levels of family emotional support (*b* = 0.422, 95% CI = 0.246, 0.682). The magnitude of the indirect association was more positive. Regarding extracurricular activities, at low family emotional support (*b* = −0.005, 95% CI −0.012, −0.001), moderate family emotional support (*b* = −0.008, 95% CI =−0.015, −0.002), and high family emotional support (*b* = −0.010, 95% CI = −0.020, −0.002) and all were statistically significant as postulated in H3b. The magnitude of the indirect association was more negative. In all, the results support hypotheses 1–3.

## 4. Discussion

This study examined the conditional role of family emotional support in buffering the association between school absenteeism among adolescents experiencing elevated levels of social anxiety at the high school level. We provide valuable insights into how family emotional support can lessen the consequences of social anxiety on outcomes related to school experiences (i.e., school satisfaction, school absenteeism, and extracurricular activity). The estimated models supported H1, the negative association between social anxiety and school satisfaction. Regarding H2, we found an indirect association between social anxiety, on the one hand, and school absenteeism and extracurricular activities, on the other hand, via perceived levels of school satisfaction. Also, we found evidence to support H3, where family emotional support moderated the indirect association between social anxiety and school-related outcomes through school satisfaction. Thus, these results suggest that while positive school satisfaction is associated with less absenteeism, for socially anxious adolescents, this individual situation can reduce their school satisfaction levels, which in turn is positively associated with absenteeism.

Moreover, the modest study finding aligns with the stage-environment fit theory, which suggests that adaptation is more likely if changes within the individual are matched with supportive change within the adolescent’s three primary environments: home, peer, and school [20,21,41]. Thus, the study finding suggests that where adolescents perceive the school and home context meets their psychological needs, it can positively be associated with their school attendance/-related activities. Therefore, school satisfaction and family emotional support are essential to school adjustment because pupils’ interest and positive attitudes about school will likely influence their motivation and appreciation for school-related activities [33,60].

The findings also show that, for some socially anxious adolescents who report higher levels of family emotional support, it may be beneficial in terms of reduced avoidance behaviors. Although the variance proportion was smaller than the theory suggests, it is in line with prior narrative and meta-analytic reviews that drew similar conclusions [61,62]. Indeed, Ableson [63] also pointed out that a smaller proportion of the variance should not be disregarded, especially when there is a potential for cumulative effects, as they could accrue across many years of schooling. Additionally, evidence suggests that the effect of missing school is detrimental even with or without permission, and calls for an emphasis on reducing absenteeism for any reason are growing [64,65,66]. Accordingly, the current study contributes to the sparse literature regarding why, for whom, and under what conditions school satisfaction promotes positive youth outcomes (see [18]).

Limitations of this study need to be noted. Firstly, we cannot infer causality from this cross-sectional observational study [67]. Second, the small proportion of variance in the study findings signals careful consideration of the study’s implications. Thirdly, self-report measures were used; therefore, response bias might have been present [68]. Third, we did not collect ethnicity or socio-economic status data [69]. Likewise, how Norwegian culture might have impacted the findings was not appraised [70]. Future research can explore the variables studied and the relevant demographic data. However, a strength of this study is that registered data on absenteeism was linked with the Young-HUNT3 survey data and, thus, reduces the potential problem of common-method variance by design [71].

Further, the current study includes a large population-based cohort with objective school absenteeism information collected prospectively from national administrative records, ensuring minimal recall bias risk, although reporting bias may have occurred. Lastly, all authors are mental health researchers [72]. Interpreting the results from different specialists, such as educators, might have given unexplored insights.

### Implication for Theory and Practice

These findings highlight the importance of school and home factors in social exclusion/inclusion among adolescents with SA and pave the way for future causally focused study designs investigating mechanisms underlying school conditions/classroom climate and positive youth outcomes. Previous work has generally focused on social anxiety or school-related factors, and these findings emphasize the need for further study of the interaction among these dimensions. Thus, beyond the need to intervene early in detecting and treating social anxiety symptoms, the results also suggest that a shared school–family–community agenda can be essential to bring mental health promotion, prevention, and intervention to families and students as part of a multitiered system of support. Notably, adolescents with social anxiety will likely not present themselves for assessment and treatment for various reasons. The findings of Yu et al. [73] support that a person-centered approach that considers the heterogeneity of social anxiety is appropriate for understanding and studying the phenomenon, as it takes greater account of differences between adolescents and suggests that moderate levels of cognitive disturbance can be adaptive. That could be done by promoting approach coping strategies and social skills in schools and families [74,75].

Moreover, positive relationships that support adolescent adaptiveness are characterized by consistency, trust, care, and responsiveness [75,76,77] and contribute to feelings of connectedness, a sense of agency, and the ability to regulate emotions, cognition, and behavior [76,78]. Overall, these findings have implications for the sustainable development goals (SDGs) to safeguard healthy lives and promote well-being for all (SDG3) and to promote inclusive and equitable quality education and the promotion of lifelong learning opportunities for all (SDG4). Parents, educators, and mental health professionals, therefore, need to tailor their approaches to address the specific challenges associated with adolescents experiencing social anxiety.

## 5. Conclusions

The modest scope of this study suggests a set of interrelations between adolescents’ perceived social anxiety, school satisfaction, family emotional support, and school absenteeism. Overall, our study’s hypothesized model was statistically supported and contributed to the discourse of the shared school–family–community in shaping students’ mental health promotion, prevention, and intervention and its association with school-related outcomes (e.g., absenteeism/extracurricular activities) among socially anxious pupils.

## Figures and Tables

**Table 1:** Descriptive statistics: mean, standard deviation, and distribution (%).

Variable	N (%)	
Sex		
Girls	1063 (57%)	
Boys	801 (43%)	
Class level (%)		
Year 1	658 (35.3%)	
Year 2	755 (40.5%)	
Year 3	451 (24.2%)	
Family financial situation (%)		
Equal	1281 (68.7%)	
Better	416 (22.3%)	
Worse	167 (9.0%)	
Self-reported health status (%)		
Not good	201 (10.8%)	
Good	993 (53.3%)	
Very good	670 (35.9%)	
Main variables	Mean (SD)	Range
Social anxiety	1.96 (0.72)	1–5
School satisfaction	2.93 (0.49)	1–4
School absenteeism	7.55 (7.62)	1–100
Family emotional support	1.79 (0.83)	1–5

**Table 2:** Bivariate association of covariates of high school absenteeism.

Variables	(1)	(2)	(3)	(4)	(5)	(6)	(7)	(8)	(9)
(1) School absenteeism	1.000								
(2) Extracurricular activities	−0.021	1.000							
(3) Social anxiety score	0.055 *	−0.097 *	1.000						
(4) Family emotional support	−0.098 *	0.105 *	−0.280 *	1.000					
(5) School satisfaction	−0.175 *	0.116 *	−0.250 *	0.273 *	1.000				
(6) Sex	−0.066 *	0.040	−0.212 *	0.034	0.010	1.000			
(7) Class level	0.035	−0.014	0.033	0.006	0.013	−0.017	1.000		
(8) Self-reported health status	−0.118 *	0.232 *	−0.294 *	0.283 *	0.233 *	0.166 *	−0.012	1.000	
(9) Self-reported family financial status	0.032	0.032	0.026	−0.094 *	0.011	0.097 *	−0.030	−0.033	1.000

*** *p* < 0.001, ** *p* < 0.01, * *p* < 0.05.

**Table 3:** Estimated path coefficients of the moderated-mediation model of social anxiety on school outcomes.

Predictors	School Satisfaction	School Absenteeism	Extracurricular Activities
	(Mediator)	(Outcome)	(Outcome)
***Control variables***			
Sex (female = 0; male = 1)			
Male	−0.069 ** (0.03)	−0.975 ** (0.35)	−0.007 (0.02)
Family financial situation			
Equal (reference category)			
Better	0.078 ** (0.03)	0.593 (0.43)	0.064 * (0.03)
Worse	0.028 (0.05)	0.604 (0.54)	0.023 (0.05)
Self-reported health (reference good)			
Good (reference category)			
Not good	−0.108 ** (0.04)	1.898 * (0.67)	−0.172 *** (0.04)
Very good	0.117 *** (0.12)	−0.392 (0.41)	0.165 *** (0.03)
Class-level			
Year-2 (reference category)			
Year-1	−0.025 (0.03)	0.003 (0.37)	0.014 (0.03)
Year-3	0.006 (0.03)	0.803 (0.49)	0.001 (0.03)
***Independent variables***			
Social Anxiety Score (SA)	−0.138 *** (0.02)	−0.222 (0.260)	−0.014 (0.02)
Family emotional support (FMS)	0.127 *** (0.02)	-	-
School satisfaction	-	−2.336 *** (0.44)	0.055 * (0.02)
SA X FMS	−0.055 * (0.03)	-	-
R^2^	0.135	0.045	0.062

Note: Standard errors are presented in parentheses; Bold values indicate statistically significant results: *** *p* < 0.001, ** *p* < 0.01, * *p* < 0.05.

**Table 4:** Conditional indirect effects of family emotional support (PFS) on the relationship between social anxiety and school outcomes via school satisfaction.

Conditional Indirect Effects	School Absenteeism		Extracurricular Activities	
	Coeff.	95% CI	Coeff.	95% CI
Low PFS	0.216	[0.098, 0.388]	−0.005	[−0.012, −0.001]
Moderate PFS	0.322	[0.193, 0.500]	−0.008	[−0.015, −0.002]
High PFS	0.422	[0.246, 0.682]	−0.010	[−0.010, −0.002]
***Index of moderated mediation***	0.128	[0.019, 0.278]	−0.003	[−0.008, −0.000]

## Data Availability

Given the legislation governing the use of the HUNT dataset and population registry data provided by Statistics Norway, our data cannot be publicly available. However, the data supporting this study’s findings are available on request per the agreement with the owner of the data, the HUNT Research Centre and Statistics Norway, and the approver of the research, the Regional Committees for Medical and Health Research Ethics (REC).
